# Clinical and Epidemiological Characteristics of Adult Patients With Primary Brain Tumors in Guatemala: A Retrospective Study

**DOI:** 10.7759/cureus.108672

**Published:** 2026-05-11

**Authors:** Luis Alejandro Echeverría Orozco

**Affiliations:** 1 General Practice, Faculty of Medicine, Universidad Mesoamericana, Quetzaltenango, GTM

**Keywords:** brain neoplasms, cancer epidemiology, cancer in latin america, guatemala, primary brain tumors, retrospective studies

## Abstract

Introduction: Brain tumors cause high morbidity and mortality and decrease patients' quality of life and autonomy. While their clinical and epidemiological characteristics have been described worldwide, data are scarce in resource-limited areas, where diagnosis and treatment are often difficult.

Purpose: This study aimed to characterize the clinical and epidemiological profile of patients with primary brain tumors aged 20-65 years admitted to the Neurosurgery Department of the Regional Hospital of the West (Guatemala) between January 2018 and January 2022, and, as exploratory secondary aims, to assess the associations between sex and tumor classification, World Health Organization grade and postoperative complications, and tumor classification and postoperative functional autonomy.

Methods: A retrospective, single-center observational study was conducted with descriptive analyses and exploratory inferential testing. The full cohort (n = 28) was analyzed for epidemiological and clinical variables; analyses of discharge status and postoperative functional autonomy (Karnofsky Performance Status (KPS)) were restricted to 23 patients, after excluding five who left against medical advice before hospital discharge. KPS was retrospectively documented at discharge from clinical descriptors in the medical record. Categorical variables are presented as frequencies, n (%), and continuous variables as mean ± standard deviation or median (interquartile range). Missing data were handled case-wise. Analyses were performed using Microsoft Excel 2024 (Microsoft Corporation, Redmond, WA) and Python 3.11 with SciPy (Python Software Foundation, Fredericksburg, VA; Fisher's exact and Mann-Whitney U tests; p < 0.05).

Results: It was found that 57.2% (n = 16) of tumors occurred in patients aged 51-65 years, 53.6% (n = 15) were female, 46.4% (n = 13) were from Quetzaltenango, and 71.4% (n = 20) were Ladino/Mestizo. Meningiomas accounted for 46.4% (n = 13); mortality was 26.1% (n = 6). Exploratory analyses showed nonsignificant trends: higher malignancy in men (risk ratio (RR) = 1.54, 95% confidence interval (CI) = 0.42-5.64; p = 0.670), more postoperative complications with high-grade tumors (RR = 1.68, 95% CI = 0.62-4.58; p = 0.603), and lower postoperative KPS in malignant vs. benign tumors (median 30 vs. 70; Mann-Whitney U = 60.5; p = 0.256).

Conclusion: Primary brain tumors in this cohort were more frequent in female patients, with meningiomas as the most common histopathology and most patients residing in the hospital's department. Unlike studies typically reporting median ages above 70 years, patients here were younger (51-65 years) with a higher mortality rate. Exploratory analyses showed nonsignificant trends consistent with the international literature; given the descriptive, retrospective, single-center design and small sample size, these findings should be interpreted as hypothesis-generating only and do not support causal inference. No prior Guatemalan study reported prevalence by ethnic group, establishing a precedent for future research.

## Introduction

Primary brain tumors constitute approximately 1.3% of all neoplasms in adults, affecting an estimated 180,047 individuals in 2020, according to statistical data from the United States. These tumors represent a significant source of morbidity, with profound psychosocial and economic repercussions for affected patients. Additionally, they are associated with a five-year mortality rate of 33.8%. Advances in therapeutic approaches have led to the continuous refinement of neurosurgical and medical treatment modalities, which are increasingly tailored based on specific tumor-related genetic markers and biomarkers. However, some of these treatments remain under investigation in ongoing clinical trials [1].

Given the need for further epidemiological and clinical characterization, a retrospective descriptive study was conducted using patient records from individuals diagnosed with primary brain tumors aged 20-65 years, who underwent neurosurgical intervention at the Regional Hospital of the West in Quetzaltenango, Guatemala, between January 2018 and January 2022. The study population comprised 28 patients, of whom 23 were included in the final sample using nonprobabilistic sampling.

The primary objective of this study was to characterize the clinical and epidemiological profile of adult patients with primary brain tumors who underwent neurosurgical treatment at the Regional Hospital of the West, including age, sex, geographic origin, ethnicity, tumor location, histopathological diagnosis, World Health Organization (WHO) tumor grade (2007 classification), Simpson grade of resection for meningiomas, postoperative complications, postoperative functional autonomy (Karnofsky Performance Status (KPS)), and discharge status. As prespecified secondary, exploratory objectives, the study examined three associations of clinical interest: 1) tumor classification (benign vs. malignant) and sex, 2) WHO tumor grade and the occurrence of postoperative complications, and 3) tumor classification and postoperative KPS score. Given the sample size, all inferential analyses were considered exploratory and hypothesis-generating.

This study contributes valuable epidemiological and clinical data on primary brain tumors in this resource-limited setting, where such information was previously unavailable. The findings allow comparison with international and prior national research, contextualizing the condition of affected patients within the Guatemalan healthcare landscape.

This study is based on an unpublished thesis by the author (Unpublished Data: Evolution of Patients Diagnosed With Primary Brain Tumors Undergoing Neurosurgical Treatment, Aged 20 to 65, Admitted to the Neurosurgery Service of the Western Regional Hospital Between January 2018 and January 2022; 2024. Universidad Mesoamericana, Quetzaltenango, Guatemala), which is being archived in the institutional repository of Universidad Mesoamericana, Quetzaltenango, Guatemala. The data presented here are a revised and corrected version of the original thesis, incorporating five additional patients and adding inferential analyses.

## Materials and methods

A retrospective, single-center, descriptive study was conducted at the Neurosurgery Department of Hospital Regional de Occidente (Quetzaltenango, Guatemala), including all adult patients who met the inclusion criteria detailed below. A nonprobabilistic, consecutive sampling technique was used to include every eligible patient. The full cohort comprised 28 patients; analyses of postoperative functional autonomy (KPS) and discharge status were restricted to 23 patients after excluding five who left against medical advice before hospital discharge, for whom a discharge KPS could not be retrospectively documented. These five patients remained included in all other analyses, and the potential selection bias introduced by their exclusion is acknowledged in the Limitations section. Given the small sample size, all inferential analyses were considered exploratory and hypothesis-generating.

Selection criteria

Inclusion Criteria

The study included patients with primary brain tumors diagnosed by tumor biopsy with histopathological analysis at the hospital's Pathology Department, patients aged 20-65 years, admitted to the Neurosurgery Department of Hospital Regional de Occidente between January 2018 and January 2022, and who received neurosurgical treatment.

Exclusion Criteria

The study excluded patients with metastatic brain tumors, primary brain tumors not treated surgically, and those with a lack of a tumor biopsy.

Data collection and processing

Biopsy records were retrieved from the Pathology Department and matched to each patient's clinical record from the Medical Records Department. Variables were entered into a Google Forms (Google LLC, Mountain View, CA) template and grouped as follows.

Epidemiological Variables

The epidemiological variables included age, sex, department of origin, and ethnic group.

Clinical Variables

They included tumor location, histopathological diagnosis, WHO tumor grade (2007 Classification of Tumors of the Central Nervous System [2]; this edition was used because access to molecular diagnostics is limited at this institution), Simpson grade of resection for meningiomas [3], type of surgical treatment, postoperative complications, postoperative KPS [4], and discharge status.

The dataset from the author's original thesis was reviewed and expanded to include five additional patients not considered in the initial analysis.

The postoperative KPS score was documented retrospectively by the principal investigator at the time of hospital discharge after the index neurosurgical admission, using the clinical descriptors recorded in each patient's medical record (physician progress notes, nursing notes, and functional descriptors documented in the discharge summary). KPS at discharge is a recognized prognostic time point in retrospective brain tumor cohorts [5]. For patients who died during the index hospitalization, KPS = 0 was assigned at the time of in-hospital death. The thesis advisor independently reviewed the KPS scores. This retrospective chart-based KPS assessment approach is supported by published methodological work [6-8].

To minimize transcription and classification errors, every record was independently reviewed by the principal investigator and thesis advisor against the biopsy report and medical record. Missing data were handled case-wise: affected cases were excluded only from analyses involving the missing variable and retained in all others. Specifically, one Simpson grade was not described in the medical record (Table 1); one WHO grade was not documented, and eight tumors were of types not graded by the WHO scale (Table 2); and five patients were excluded from the KPS and discharge-status analyses (Tables 3-5).

**Table 1:** Grade of excision of meningiomas Data are represented as frequencies (n) and percentages (%); analysis restricted to patients with meningiomas (n = 13). The term "not described" was used in one case, for which the medical records did not indicate the type of excision performed

Grade of excision	n (%)
Simpson I	6 (46.2)
Simpson V	3 (23.1)
Simpson II	2 (15.4)
Simpson IV	1 (7.7)
Not described	1 (7.7)
Simpson III	0 (0)
Grand total	13 (100)

**Table 2:** WHO tumor grade Data are represented as frequencies (n) and percentages (%). Among gradable tumors (n = 19): low grade (I + II) = 14 (73.7%); high grade (IV) = 5 (26.3%) WHO: World Health Organization

Tumor grade	n (%)
Low grade (I-III)	14 (73.7)
High grade (IV)	5 (26.3)
Total	19 (100)

**Table 3:** Discharge status Data are represented as frequencies (n) and percentages (%). Analytic sample restricted to 23 patients; five patients who requested discharge against medical advice before the standardized assessment were excluded from this analysis, but were retained in all other analyses

Discharge status	n (%)
Survived	17 (74)
Deceased	6 (26)
Total	23 (100)

**Table 4:** KPS score Data are represented as frequencies (n) and percentages (%); KPS scores: mean ± SD = 57.4 ± 38.5; median (IQR) = 70 (15-90). Analytic sample restricted to 23 patients; five patients who requested discharge against medical advice before the standardized KPS assessment were excluded from this analysis KPS: Karnofsky Performance Status; SD: standard deviation; IQR: interquartile range

KPS score	n (%)
100	5 (21.7)
90	3 (13.0)
80	2 (8.7)
70	3 (13.0)
60	3 (13.0)
30	1 (4.3)
0	6 (26.1)
Total	23 (100)

**Table 5:** Comparison of postoperative KPS score between patients with benign and malignant tumors Data are presented as median and IQR, and as mean ± SD. KPS scores ranged from 0 to 100 across all groups. The test statistic is the Mann-Whitney U value. The p value was calculated using the Mann-Whitney U test, selected as a nonparametric alternative appropriate for small samples and ordinal data with nonnormal distribution. Significance threshold was set at p < 0.05. Analytic sample (n = 23) restricted to patients with a standardized postoperative KPS assessment; five patients who requested discharge against medical advice before assessment were excluded from this analysis ^*^Benign tumors: meningioma, pituitary adenoma, oncocytoma, and schwannoma ^†^Malignant tumors: glioblastoma, astrocytoma, and oligodendroglioma KPS: Karnofsky Performance Status; SD: standard deviation; IQR: interquartile range

Tumor classification	n	Median (IQR)	Mean ± SD	Test statistic	p value
Benign^*^	18	70 (60-90)	62.8 ± 36.8	U = 60.5	0.256
Malignant^†^	5	30 (0-60)	38.0 ± 42.7		
Overall	23	70 (15-90)	57.4 ± 38.5		

Categorical variables are presented as frequencies (n) and percentages (%); continuous variables as mean ± standard deviation or median (interquartile range, IQR). Data were tabulated in Microsoft Excel 2024 (Microsoft Corporation, Redmond, WA). Inferential analyses were performed in Python 3.11 (Python Software Foundation, Fredericksburg, VA) with the SciPy library (scipy.stats), using Fisher's exact test for categorical comparisons (selected over the chi-square test because expected cell counts were <5) and the Mann-Whitney U test for ordinal comparisons with nonnormal distribution. Statistical significance was set at p < 0.05.

## Results

Epidemiological profile

Table 6 shows that nearly half of the patients in the sample fall within the age groups of 51-55, 61-65, and 56-60 years (16 patients). This demonstrates that primary brain tumors were more prevalent during and after the fifth decade of life, representing 57.2% of the sample.

**Table 6:** Distribution based on age groups Data are represented as frequencies (n) and percentages (%). For continuous interpretation, age (years): mean ± SD = 49.4 ± 11.5; median (IQR) = 53 (43-58); range = 28-63 SD: standard deviation; IQR: interquartile range

Age groups (years)	n (%)
51-55	6 (21.4)
61-65	5 (17.9)
56-60	5 (17.9)
26-30	3 (10.7)
46-50	3 (10.7)
41-45	3 (10.7)
31-35	2 (7.1)
36-40	1 (3.6)
Total	28 (100)

Likewise, in this study, female patients exhibited a higher prevalence of the disease (54%, corresponding to 15 patients) than male patients (46%, corresponding to 13 patients), with a ratio of 1.15:1, as shown in Table 7.

**Table 7:** Sex distribution Data are represented as frequencies (n) and percentage (%). The female-to-male ratio is 1.15:1

Sex	n (%)
Female	15 (53.6)
Male	13 (46.4)
Total	28 (100)

According to Table 8, nearly half of the subjects were from the department of Quetzaltenango, where the aforementioned hospital is located (13 patients, representing the 46.4% of the sample).

**Table 8:** Distribution based on the department of origin Data are represented as frequencies (n) and percentages (%). The term "department" refers to an administrative division within Guatemala, equivalent to "province" in other countries

Department of origin	n (%)
Quetzaltenango	13 (46.4)
San Marcos	4 (14.3)
Huehuetenango	3 (10.7)
Totonicapan	3 (10.7)
Suchitepequez	2 (7.1)
Retalhuleu	2 (7.1)
Solola	1 (3.6)
Total	28 (100)

In this study, the majority of patients were part of the Ladino/Mestizo ethnic group (20 patients, 71.4%), while the rest were from the Maya ethnic group (eight patients, 28.6% of the sample). No data were recorded regarding people from the other two ethnic groups in Guatemala (Garífuna and Xinca), shown in Table 9.

**Table 9:** Distribution based on the ethnic group Data are represented as frequencies (n) and percentages (%). Ladino: a term used in Guatemala to describe people of mixed Indigenous and European descent who have adopted a Hispanic culture [9]. Mestizo: a general term in Latin America referring to individuals of mixed Indigenous and European ancestry [10]. Maya: Indigenous people descended from the ancient Maya civilization [11]. Garífuna and Xinca: the two remaining major ethnic groups in Guatemala, not represented in this sample

Ethnic group	n (%)
Ladino/Mestizo	20 (71.4)
Maya	8 (28.6)
Total	28 (100)

Clinical profile

Diagnosis

In this sample, the most common tumor location was the meninges, affecting 46.4% of patients (13 cases), followed by the frontal lobe (17.9%, five cases) and the sellar region (14.3%, four cases). Tumors were also found in the parietal lobe (10.7%, three cases). Less frequently, tumors were identified in the frontotemporoparietal, temporoparietal, and frontoparietal regions, each accounting for 3.6% (one case) (Table 10).

**Table 10:** Distribution based on the tumor location Data are represented as frequencies (n) and percentages (%)

Tumor location	n (%)
Meninges	13 (46.4)
Frontal	5 (17.9)
Sellar region	4 (14.3)
Parietal	3 (10.7)
Frontotemporoparietal	1 (3.6)
Temporoparietal	1 (3.6)
Frontoparietal	1 (3.6)
Total	28 (100)

It was found that 46.4% of tumors in the studied patients corresponded to meningiomas (13 patients). Pituitary adenomas account for 17.9% (five patients). A total of 10.7% of patients have been diagnosed with glioblastomas or astrocytomas (three patients in each tumor group). Oncocytomas represents 7.1% of cases (two patients). Finally, schwannomas and oligodendrogliomas have the lowest prevalence, with one patient each, equivalent to 3.6% each of the total patients, as indicated in Table 11.

**Table 11:** Distribution based on the histopathology of the brain tumors Data are represented as frequencies (n) and percentages (%)

Tumor type	n (%)
Meningioma	13 (46.4)
Pituitary adenoma	5 (17.9)
Glioblastoma	3 (10.7)
Astrocytoma	3 (10.7)
Oncocytoma	2 (7.1)
Schwannoma	1 (3.6)
Oligodendroglioma	1 (3.6)
Total	28 (100)

Among the 28 patients in the cohort, eight (28.6%) had tumor types not graded according to the WHO scale (primarily benign tumors such as pituitary adenomas, oncocytomas, and schwannomas), and one (3.6%) had a grade not documented in the clinical record. Among the 19 patients with a documented WHO grade, 14 (73.7%) had low-grade tumors (grades I-III), and five (26.3%) had high-grade tumors (grade IV), as shown in Table 2.

Treatment

Regarding the procedures performed in patients with meningiomas, the extent of resection was classified according to the Simpson grading system [3]. A total of 46.2% underwent Simpson Grade I excision, achieving complete tumor resection. Simpson Grade V resection was performed in 23.1% of cases, indicating a more conservative surgical approach. Simpson Grade II excision, which involves near-total resection, was observed in 15.4% of patients, while 7.7% underwent Simpson Grade IV excision, representing a more limited resection. No cases corresponding to Simpson Grade III were identified. These findings are summarized in Table 1.

As shown in Table 12, complications were absent in 18 patients (64.3%), while 10 patients (35.7%) experienced at least one complication. Neuroinfectious complications were observed in seven patients (25.0%), including three cases (10.7%) in which they occurred in isolation and one case (3.6%) in association with seizures. Mortality was recorded in six patients (21.4%), comprising three cases (10.7%) as an isolated outcome and three cases (10.7%) in which death occurred in conjunction with neuroinfection. Overall, neuroinfection-related complications accounted for 25.0% of the total sample.

**Table 12:** Postoperative complications Data are represented as frequencies (n) and percentages (%). Overall mortality rate = 6/28 (21.4%); any neuroinfection = 7/28 (25.0%)

Postoperative complications	n (%)
None	18 (64.3)
Death	3 (10.7)
Neuroinfections	3 (10.7)
Neuroinfections and death	3 (10.7)
Neuroinfections and seizures	1 (3.6)
Total	28 (100)

*Discharge and Prognosis*

The analysis of discharge status and postoperative functional autonomy was restricted to 23 patients (the analytic sample), after excluding five patients who requested discharge against medical advice before a standardized KPS assessment could be performed. All other analyses in this study were conducted on the full cohort of 28 patients. According to the data presented in Table 3, of the total sample (n = 23), 74% (17 patients) were discharged alive, while 26% (six patients) did not survive.

Table 4 presents the distribution of patients’ scores according to the KPS [4] at discharge, highlighting their functional status. A total of 21.7% of patients (n = 5) achieved a KPS score of 100. Additionally, 13% (n = 3) scored 90, 8.7% (n = 2) scored 80, and 13% (n = 3) scored 70. Another 13% (n = 3) had a KPS score of 60, while 4.3% (n = 1) scored 30. Finally, 26.1% of patients (n = 6) had a KPS score of 0.

Associations

Table 13 presents the association between tumor classification and sex among the 28 patients in the cohort. Among female patients (n = 15), 80.0% (n = 12) had benign tumors, and 20.0% (n = 3) had malignant tumors. Among male patients (n = 13), 69.2% (n = 9) had benign tumors, and 30.8% (n = 4) had malignant tumors. Patients with malignant tumors represented 25.0% (n = 7) of the total cohort. Male patients had 1.54 times the risk of presenting with a malignant tumor compared with female patients (risk ratio (RR) = 1.54; 95% confidence interval (CI) = 0.42-5.64), although this association did not reach statistical significance (χ² = 0.43; Fisher's exact test, p = 0.670).

**Table 13:** Association between tumor classification (benign vs. malignant) and sex Data are presented as frequencies (n) and percentages (%); column percentages within each sex. Effect size is reported as RR with 95% confidence interval. The test statistic is the chi-square value (χ²). The p value was calculated using Fisher's exact test, selected over the chi-square test because the minimum expected cell count was 3.25 (<5), which violates the assumption underlying the chi-square approximation. Significance threshold set at p<0.05 ^*^Benign tumors: meningioma, pituitary adenoma, oncocytoma, schwannoma ^†^Malignant tumors: glioblastoma, astrocytoma, oligodendroglioma ^‡^RR for the risk of malignancy in men compared with women RR: risk ratio; CI: confidence interval

Tumor classification	Female, n (%)	Male, n (%)	Total, n (%)	Effect size (95% CI)	Test statistic	p value
Benign^*^	12 (80.0)	9 (69.2)	21 (75.0)	RR = 1.54 (0.42-5.64)^‡^	χ² = 0.43	0.670
Malignant^†^	3 (20.0)	4 (30.8)	7 (25.0)			
Total	15 (100)	13 (100)	28 (100)			

Table 14 presents the association between postoperative complications and WHO tumor grade in the 19 patients with a documented WHO grade; eight patients with nonapplicable grading and one patient with missing grade information were excluded from this analysis. Among patients with low-grade tumors (WHO I-III, n = 14), 64.3% (n = 9) had no postoperative complications, while 35.7% (n = 5) developed at least one complication. Among patients with high-grade tumors (WHO IV, n = 5), 40.0% (n = 2) had no complications, and 60.0% (n = 3) developed at least one complication. Patients with high-grade tumors had 1.68 times the risk of developing a postoperative complication compared with those with low-grade tumors (RR = 1.68, 95% CI = 0.62-4.58), although this association did not reach statistical significance (χ² = 0.89; Fisher's exact test, p = 0.603).

**Table 14:** Association between postoperative complications and WHO tumor grade Data are presented as frequencies (n) and percentages (%); column percentages within each WHO grade group. Effect size is reported as RR with 95% confidence interval. The test statistic is the chi-square value (χ²). The p value was calculated using Fisher's exact test, selected over the chi-square test because the minimum expected cell count was 2.11 (<5), which violates the assumption underlying the chi-square approximation. Significance threshold set at p < 0.05. Analytic sample (n = 19) restricted to patients with a documented WHO grade; tumors classified as “not applicable” (n = 8, primarily benign tumors not graded by the WHO scale) and “not described” (n = 1, missing in clinical records) were excluded from this analysis ^†^RR for the risk of any complication in patients with high-grade tumors compared with those with low-grade tumors RR: risk ratio; CI: confidence interval; WHO: World Health Organization

Complications	Low grade (I-III), n (%)	High grade (IV), n (%)	Total, n (%)	Effect size (95% CI)	Test statistic	p value
No complications	9 (64.3)	2 (40.0)	11 (57.9)	RR = 1.68 (0.62-4.58)^†^	χ² = 0.89	0.603
Any complication	5 (35.7)	3 (60.0)	8 (42.1)			
Total	14 (100)	5 (100)	19 (100)			

Table 5 presents the comparison of postoperative KPS scores between patients with benign and malignant tumors among the 23 patients with a standardized postoperative assessment; five patients who requested discharge against medical advice before assessment were excluded. Patients with benign tumors (n = 18) achieved a median KPS score of 70 (IQR = 60-90), with a mean of 62.8 ± 36.8. Patients with malignant tumors (n = 5) had a substantially lower median KPS score of 30 (IQR = 0-60), with a mean of 38.0 ± 42.7. The overall median for the analytic sample was 70 (IQR = 15-90), with a mean of 57.4 ± 38.5. Although patients with benign tumors showed numerically higher functional outcomes than those with malignant tumors, the difference did not reach statistical significance (Mann-Whitney U = 60.5; p = 0.256).

## Discussion

Epidemiological profile

Meningiomas were the most prevalent tumors in this cohort, consistent with the Central Brain Tumor Registry of the United States (CBTRUS) 2023, which reported a higher female incidence (27.85 vs. 21.62 per 100,000; 56.3% female vs. 43.7% male patients) [12]. In contrast, a separate U.S. study reported male predominance in glioblastomas, attributed to reduced Retinoblastoma Protein (Rb) activity in male patients [13], and a 2022 Mexican cohort of 58 adult patients similarly found male patients to be more affected (55.17%) [14]. The female predominance in the present study likely reflects the higher prevalence of meningiomas, a tumor type more common in women, and a trend potentially linked to lower androgenic load, although this association has not been fully established [15].

Regarding department of origin (Table 8), nearly half of the patients were from Quetzaltenango (46.4%; 13 patients), followed by San Marcos (14.3%; four patients), reflecting proximity to the hospital. The same pattern was observed in a thesis from Roosevelt Hospital in Guatemala City (1992-1997), in which 50.51% of patients came from the department in which the hospital is located [16].

Patients of the Ladino/Mestizo ethnic group were more affected (71.4%; 20 patients) than those of Maya ethnicity (28.6%; eight patients) (Table 9); no Garífuna or Xinca patients were recorded. No previous Guatemalan study has included this variable, but the distribution aligns with the 2021 Statistical Compendium of Peoples (63.4% Ladino, ~35% Maya) [17].

Clinical profile

Diagnosis

The most common tumor location was the meninges (46.4%, n = 13), followed by the frontal lobe (17.9%, n = 5) and the sellar region (14.3%, n = 4) (Table 10). This is consistent with CBTRUS, which identifies the meninges as the most frequent anatomical site, followed by the frontal region [18]. In contrast, two prior Guatemalan studies reported the frontal lobe as the most affected location: Roosevelt Hospital (1992-1997, 23.71%, followed by the pineal region at 21.65%) [16] and the Guatemalan Social Security Institute Hospital (1993-1998, 17% in the frontal and occipital lobes and the sellar region) [19].

Meningiomas accounted for 46.4% of cases (13 patients), followed by pituitary tumors (17.9% adenomas plus 7.1% oncocytomas) and glioblastomas (10.7%, n = 3) (Table 11). This distribution mirrors CBTRUS 2016-2020 data (40.5% nonmalignant meningiomas, 17.2% nonmalignant pituitary tumors, 14.2% glioblastomas) [12] but differs from a 2022 Mexican surgical cohort (29.5% meningiomas, 25% glioblastomas, 5.7% pituitary) [20] and from earlier Guatemalan studies: gliomas were most prevalent at Roosevelt Hospital (36.8%) [16], and meningiomas (14%) and glioblastomas (13%) predominated at the Guatemalan Social Security Institute [19]. These differences likely reflect changes in tumor distribution over the past two decades. It is also important to note that the cited Guatemalan studies relied on clinical and neuroimaging-based diagnosis, whereas CBTRUS is a histopathology-based registry that requires microscopic confirmation [12], which may partially account for the case-mix differences.

The 2007 WHO Classification of Tumors of the Central Nervous System classifies neoplasms by aggressiveness and biological behavior. Among the 19 patients with a documented WHO grade, 73.7% (n = 14) had low-grade tumors (grades I-III) and 26.3% (n = 5) had high-grade tumors (grade IV) (Table 2), consistent with the predominance of benign over malignant lesions reported in the literature, where benign tumors account for approximately 68% of primary brain tumors [21]. The 2007 edition was used because molecular profiling is not routinely available at the institution. These results differ from a 2008-2011 retrospective study reporting 44% benign and 47.5% malignant tumors, a difference likely attributable to that study's inclusion of metastatic cases [22].

Treatment

Among meningioma patients, 46.2% underwent Simpson Grade I excision (complete tumor resection), followed by Simpson Grade V (23.1%), Simpson Grade II (15.4%), and Simpson Grade IV (7.7%); no Grade III resections were identified (Table 1). This Simpson Grade V proportion contrasts with a retrospective Argentine study of Grade I meningiomas (2006-2015), in which Simpson Grade I resection was performed in 85% of convexity meningiomas (n = 67) and in 30.6% of skull base meningiomas (n = 160), with Simpson Grade II in 35.6% and Simpson Grade III in 24.4% of the latter; no Grade V resections were documented [23]. A California study likewise reported a predominance of Simpson Grade IV excisions (37%), with no Grade V cases [24].

The Simpson grading system is a key determinant of recurrence and long-term remission: Grade I resections have the lowest recurrence rate (~5%-10% at 10 years) [3]; Grade II, 20%-30% [24]; Grade III, 30%-40% [25]; Grade IV, 50%-60% at 10 years; and Grade V, near-inevitable recurrence unless followed by adjuvant therapy [26]. WHO grade also independently influences recurrence, with atypical (WHO II) and anaplastic (WHO III) meningiomas showing higher regrowth even after aggressive resection [27]. These findings underscore the importance of complete surgical excision whenever feasible.

Postoperative Complications

Most patients (64.3%, n = 18) had no postoperative complications, while 35.7% experienced at least one (Table 12). Neuroinfections (10.7%), mortality (10.7%), and the combination of both (10.7%) were equally prevalent, with seizures the least frequent. The overall neuroinfection rate (25.0%) exceeds that reported in a 2000-2022 meta-analysis of 220 studies of postoperative meningitis after elective cranial surgeries (0.1%-10%, with variability depending on the country's level of development) [28].

*Discharge and Prognosis*

The in-hospital mortality rate was 26.1% (n = 6) in the analytic sample of 23 patients with documented discharge status, and 21.4% (n = 6) in the full cohort of 28 patients (Table 3). This exceeds the 2.8% reported in a 2021 European multicenter study of 4,864 patients under 75 years [29] and the 10.6% reported in a 2014 Cuban study of high-grade gliomas [30].

Postoperative functional autonomy at discharge, assessed with the KPS [4] (Table 4), showed nearly half of patients with favorable recovery (KPS = 80-100, n = 10, 43.5%; overall mean = 57.4 ± 38.5; median = 70, IQR = 15-90). This is higher than the mean KPS of 41.1 reported in a 2008-2011 Colombian study of 78 brain tumor patients [22] but lower than the means reported in a Chinese study of 104 patients with low-grade supratentorial gliomas (mean = 75) [31] and a Croatian retrospective study of 63 glioblastoma patients (mean = 77) [32].

Inferential analyses

The first analysis evaluated the relationship between tumor classification and sex (Table 13). Among female patients, 80.0% (n = 12) had benign tumors, and 20.0% (n = 3) had malignant tumors; among male patients, 69.2% (n = 9) had benign tumors, and 30.8% (n = 4) had malignant tumors. Male patients had 1.54 times the risk of presenting with a malignant tumor compared with female patients (RR = 1.54, 95% CI = 0.42-5.64; χ² = 0.43; Fisher's exact test, p = 0.670). Although the difference did not reach statistical significance, the direction of the association is consistent with international literature reporting a higher prevalence of high-grade tumors, particularly glioblastomas, among male patients [14,15]. The wide CI and lack of statistical significance likely reflect the limited statistical power inherent to the small sample size, and this finding should be interpreted as hypothesis-generating rather than as evidence of no association.

The second analysis examined the association between WHO tumor grade and the occurrence of postoperative complications (Table 14). Among patients with low-grade tumors (WHO I-III), 35.7% (n = 5) developed at least one complication, compared with 60.0% (n = 3) of patients with high-grade tumors (WHO IV). Patients with high-grade tumors had 1.68 times the risk of developing a postoperative complication compared with those with low-grade tumors (RR = 1.68, 95% CI = 0.62-4.58; χ² = 0.89; Fisher's exact test, p = 0.603). Although this trend is biologically plausible, given that high-grade tumors are typically more invasive and may require more complex surgical approaches, the literature is inconsistent: a recent single-center study in Guinea similarly examining surgical complications in 65 brain tumor patients found that the histological type of glioma was not a significant predictor of complications on multivariate analysis (adjusted OR = 0.74, p = 0.671), although factors such as preoperative performance status, surgery duration, partial resection, and infratentorial location were significantly associated with increased morbidity [33]. As with the previous comparison, the limited sample size and the small number of patients in the high-grade subgroup (n = 5) constrain the statistical power of the present analysis.

The third analysis compared postoperative functional outcomes, measured by the KPS, between patients with benign and malignant tumors (Table 5). Patients with benign tumors had a substantially higher median KPS score at discharge (70, IQR = 60-90) than patients with malignant tumors (30, IQR = 0-60), with mean values of 62.8 ± 36.8 and 38.0 ± 42.7, respectively. Although this clinically meaningful difference did not reach statistical significance (Mann-Whitney U = 60.5; p = 0.256), the magnitude of the median difference (40 points on the KPS) is consistent with prior reports describing poorer functional recovery in patients with high-grade or malignant brain tumors [22,31,32]. The small malignant subgroup (n = 5) substantially limits the statistical power of this comparison, and confirmation in a larger multicenter study is warranted.

Taken together, the three inferential analyses demonstrated trends consistent with the international literature: a numerically higher proportion of malignancy among male patients (compared with females within the cohort), more postoperative complications in patients with high-grade tumors, and lower postoperative functional outcomes in patients with malignant tumors. Although none of these associations reached statistical significance, likely due to the limited statistical power of a single-center cohort of 28 patients, the consistency of the directional findings with established literature supports the validity of the observed patterns and provides a basis for future research with larger samples.

Limitations

This study has several limitations that should be considered when interpreting its findings. First, the analysis of postoperative functional autonomy and discharge status was restricted to 23 of the 28 patients, as five patients requested discharge against medical advice before a postsurgical follow-up encounter from which the KPS could be retrospectively documented. The reasons for these requests could not be reliably determined from the clinical records; in the local context, families frequently request early discharge of postsurgical patients in the immediate postoperative period, particularly when the patient remains under mechanical ventilation or appears clinically severe, without waiting for the true neurological outcome to manifest, even after physician counseling. This pattern reflects family decision-making dynamics and contextual healthcare factors rather than necessarily indicating worse clinical outcomes; therefore, the direction of any potential selection bias introduced by these exclusions cannot be assumed.

Second, the small sample size and single-center retrospective design limit the generalizability of the findings to other Guatemalan centers and to other resource-limited settings. Third, the small sample size considerably limited the statistical power of the inferential analyses to detect significant associations between subgroups; nonsignificant results should, therefore, be interpreted as hypothesis-generating rather than as evidence of no true association, and multivariable modeling was not feasible given the limited number of cases.

Fourth, the KPS in this study was assessed at hospital discharge, which captures functional status at a single time point in the immediate perioperative period and does not reflect longer term recovery or trajectory. Structured long-term outcomes such as long-term recurrence, overall survival, and longitudinal KPS trajectory were not uniformly available across the cohort, because outpatient follow-up duration was determined by clinical need (some patients required prolonged follow-up, while others were formally discharged from outpatient controls earlier when their postsurgical course was uneventful, in keeping with routine institutional practice). This limits comparability with cohorts that report KPS at later time points or longitudinal functional trajectories.

Fifth, the retrospective nature of the data collection is also susceptible to information bias arising from variability in the completeness of clinical documentation, as illustrated by the small number of missing values for Simpson and WHO grades described in the Methods section.

Sixth, tumor classification was based on the 2007 WHO Classification of Tumors of the Central Nervous System, without molecular markers (e.g., isocitrate dehydrogenase mutation, 1p/19q codeletion, MGMT promoter methylation), reflecting local diagnostic capacity; this limits direct comparability with contemporary cohorts that apply the 2016 and 2021 WHO classifications incorporating molecular criteria.

## Conclusions

In this single-center retrospective cohort of 28 adult patients with histopathologically confirmed primary brain tumors, the highest prevalence demographic subgroup was female patients (53.6%, n = 15), aged 51-65 years (57.2%, n = 16), residing in the department of Quetzaltenango (46.4%, n = 13), and self-identifying as Ladino/Mestizo (71.4%, n = 20). Meningeal tumors were the most frequent location (46.4%, n = 13), and meningioma was the most frequent histopathological diagnosis (46.4%, n = 13). Among gradable tumors (n = 19), low-grade lesions predominated (73.7%, n = 14). Among patients with meningiomas (n = 13), Simpson Grade I resection was achieved in 46.2%. Postoperative complications occurred in 35.7% of the cohort, with neuroinfections being the most frequent (25.0%); the in-hospital mortality rate was 21.4% (6/28) over the full cohort and 26.1% (6/23) within the analytic sample with documented discharge status. Postoperative functional autonomy at discharge was acceptable on average (mean KPS = 57.4 ± 38.5; median = 70, IQR = 15-90), exceeding the average reported in a Colombian cohort but falling short of those reported in studies from China and Croatia.

The three prespecified exploratory analyses showed directionally consistent but nonstatistically significant trends: a higher proportion of malignant tumors among male patients compared with female patients (RR = 1.54, 95% CI = 0.42-5.64; p = 0.670); a higher risk of postoperative complications among patients with high-grade tumors (RR = 1.68, 95% CI = 0.62-4.58; p = 0.603); and substantially lower postoperative KPS scores in patients with malignant tumors than in those with benign tumors (median 30 vs. 70; Mann-Whitney U = 60.5; p = 0.256). The directional consistency of these three findings with international literature supports the validity of the observed patterns and provides a basis for future multicenter research with larger samples.

Because of the descriptive, retrospective, single-center design, none of these results support causal inference, and the inferential findings should be interpreted as hypothesis-generating only. To our knowledge, this is the first Guatemalan study of primary brain tumors to report the prevalence by ethnic group and to apply the KPS to assess postoperative functional autonomy in this setting, establishing a baseline for future regional research.
